# Sex-Specific Physical Activity and Weight Status in German Schoolchildren: Interim Results from the Hand on Heart Study

**DOI:** 10.3390/children12040412

**Published:** 2025-03-26

**Authors:** Meike Schrader, Jennifer Wieprecht, Federico Morassutti Vitale, Simone Katrin Manai, Samar Shamas, Marcel Müller, Maren Baethmann, Anja Tengler, Roxana Riley, Guido Mandilaras, Nikolaus Alexander Haas, Delphina Gomes

**Affiliations:** 1 Division of Pediatric Cardiology and Intensive Care, University Hospital, LMU Munich, 81377 Munich, Germany; meike.schrader@med.uni-muenchen.de (M.S.); simone.dold@med.uni-muenchen.de (S.K.M.); maren.baethmann@tum.de (M.B.); anja.tengler@med.uni-muenchen.de (A.T.); roxana.riley@med.uni-muenchen.de (R.R.); guido.mandilaras@med.uni-muenchen.de (G.M.); nikolaus.haas@med.uni-muenchen.de (N.A.H.); 2 Institute for Medical Information Processing Biometry and Epidemiology, University Hospital, LMU Munich, 81377 Munich, Germany; samar.shamas@ibe.med.uni-muenchen.de (S.S.); mmueller@ibe.med.uni-muenchen.de (M.M.)

**Keywords:** physical activity, sex differences, sports participation, BMI, adolescents, sports clubs, Hand-on-heart-study (“Hand-aufs-Herz”)

## Abstract

Background/Objectives: physical activity (PA) in children offers lifelong benefits, yet nearly four in five children are insufficiently active. We examined sex-specific differences in sport participation by sport type and its association with weight status. Methods: in the ongoing hand-on-heart-study (“Hand-aufs-Herz”), comprehensive data on sociodemographic profiles, PA, and anthropometry were collected from 922 school-aged children, adolescents, and young adults (8–20 years) in Germany. Sex-specific differences in sport participation, sport types, and weight status were analyzed using descriptive statistics and logistic regression models. Results: based on the eligibility criteria for analysis (ages 8–18 years), 883 pupils were included (mean age 13.1 ± 2.4 years), 406 (46%) were females. Compared to females, males had a 43% higher chance of being members of a sports club and were more likely to spend more days/week on sports (44–85%). Males participated more in football, martial arts, and basketball while females favored gymnastics and volleyball. As age increased, males had a 30% higher likelihood of not being sports club members (females: 13%). Overweight/obese males had twice the risk of lacking sports memberships. The largest body mass index (BMI) difference was found in males and females participating in athletics, with −4.64 kg/m^2^ (males) and −1.99 kg/m^2^ (females) compared to their counterparts without club memberships. Conclusions: in conclusion, sports participation should be encouraged especially among females and overweight/obese males. Targeted strategies should focus on promoting inclusive and non-competitive activities that cater to their interests.

## 1. Introduction

Physical activity (PA) is vital for promoting healthy growth, improving cardiovascular health, and developing motor skills [1,2]. Children who engage in sports are more likely to continue participating in physical activities during adulthood, forming habits that contribute to long-term health and well-being [3]. Interestingly, participation in sports during childhood is linked to 148–191% increased levels of vigorous PA in adulthood [4]. Engaging in sports during childhood not only supports physical development but also plays a crucial role in the mental and emotional growth of children, enhancing self-esteem, teamwork, and discipline [5].

This early exposure to PA can potentially reduce the risk of chronic diseases such as obesity, type 2 diabetes, and cardiovascular conditions later in life [6]. A longitudinal study found that children and adolescents (3–18 years) who were persistently active had a significantly lower risk of adult clustered cardiometabolic risk (33% reduction), high glucose levels (23% reduction), low HDL cholesterol levels (22% reduction), obesity (24% reduction), high waist circumference (18% reduction), high insulin levels (42% reduction), and high triglyceride levels (40% reduction) compared to those who were persistently inactive, highlighting the long-term benefits of sustained PA [7].

To promote consistent PA from a young age, PA recommendations aim to establish healthy habits. Guidelines on PA for children are designed to help caretakers, schools, and communities to promote PA, ensuring children lead active lives and develop essential physical and social skills [8]. The World Health Organization (WHO) defines PA as any bodily movement produced by skeletal muscles that requires energy expenditure [8,9] and recommends that children aged 5–17 engage in at least 60 min of moderate-to-vigorous PA daily, including activities that strengthen muscles and bones at least three times a week [9]. In Germany, children aged 4–6 years should engage in 180 min of PA daily, while those aged 6–11 years and 12–18 years should aim for at least 90 min of moderate-to-vigorous activity, with 60 min from daily activities like walking 12,000 steps [10].

Despite the presence of PA guidelines in children, over 80% of school-going adolescents aged 11–17 years do not meet the WHO recommendations for daily PA, putting their current and future health at risk [11]. Evidence from Europe shows regional variations, with Italy, France, and Portugal having the highest proportions of adolescents (up to 96%) who do not engage in moderate-to-vigorous PA daily [12]. In Germany, the Global Matrix 4.0 grading rubric [13] assigned a grade of D– to adolescents for their overall PA levels, indicating that only one in four (20–26%) meet the global PA recommendations [14].

Research gaps remain in understanding the sex differences in adolescent PA, since a French study of 8084 adolescents [15] and data from the International Children Accelerometry Database (ICAD) involving 15,461 individuals across nine countries [16] revealed that boys were fitter, engaged more in sports club activities, and exhibited higher levels of moderate-to-vigorous PA, with greater variability, while no significant sex differences were found in sedentary or light-intensity activity. Sex-specific differences in PA are also apparent on weight outcomes as higher PA levels in boys are linked to lower rates of overweight and obesity, while girls’ lower PA participation is associated with higher rates of overweight and obesity [17].

Therefore, differences between sexes need to be studied further in order to explore participation rates for a variety of sports over time and how these preferences evolve with age. Furthermore, it is important to identify which specific sports are related to better weight outcomes in males and females because different sports offer varying levels of physical intensity, endurance, and muscle engagement, which can influence weight management.

Using the comprehensive hand-on-heart-study (“Hand-aufs-Herz”), we aimed to evaluate the prevalence of PA among school-going males and females. Our analysis focused on the engagement in various types of sports and compared participation rates between the sex groups. Additionally, we assessed sex-specific weight status in relation to the different sport types, examining how certain activities may impact the weight outcomes of both boys and girls. By considering both participation levels and weight status, our study provides valuable insights into the role of sports in children’s health across different sex groups.

## 2. Materials and Methods

### 2.1. Study Design and Participants

The hand-on-heart-project (German clinical trial register DRKS00033999) is an ongoing study of school-aged children, adolescents, and young adults (8–20 years). The study project aims to develop a reliable, effective, and comprehensive cardiovascular screening test that is simple to perform and cost-effective. Each participant completes a detailed questionnaire, undergoes a physical examination, the collection of biometric data and vital parameters, an ECG, an echocardiography, the determination of physical strength and a standardized fitness test. The goal is to establish this test as a preventive measure in both general and pediatric practices, as well as in clinics, and eventually integrate it into routine healthcare.

The study is based on data from the Orlando Heart Health Weeks questionnaires, which are named after the school where the research was conducted. This school was the first to take part in the hand-on-heart project, a prevention initiative by the Department of Pediatric Cardiology at LMU, supported by the Nicolas May Foundation [18]. The children, adolescents, and young adults (ages 8 to 20 years) from the selected school were allowed to take part in the project on a voluntary basis. The majority of the approximately 1000 students took up the offer. Ethical approval for the study was granted on 15 April 2024 (project number 24-0147) by the Ethics Committee of Ludwig-Maximilians-Universität.

Initially, the project was introduced to parents via a two-sided letter and informational flyers for the school management and teachers. This was followed by a detailed parents’ evening. The project was then presented to all students in their respective classes, with registration completed using a written form. The consent form contains a detailed information leaflet explaining the examination process over six pages. The consent of parents and children was required for participation.

Comprehensive data on health, PA, fitness, and nutrition is collected using a REDCap based survey that was designed for the project by our department. The pupils completed the questionnaire on a tablet on site. Information on the current state of health, daily step count, daily PA, membership to sports club and type of sports club, frequency of attending sports club per week, eating habits, and risk behavior was requested. For the evaluation of nutritional behavior, we use the published KidMed 2.0 score [19]. The included items with the corresponding answer options are listed in the Section 3.

### 2.2. Inclusion Criteria for Analysis

All children and adolescents aged between 8 and 18 years were included in the present study. Young adults (ages 18 to 20 years) were excluded from this analysis. In this manuscript, we refer to the study population as “pupils”, which encompasses the children and adolescents involved in the study.

### 2.3. Exposure Variables

Sex was used as the main exposure variable. In order to assess the role of sport activities on body mass index (BMI in kg/m^2^), sport club membership and type of sport were also considered as exposure variables.

### 2.4. Outcome Variables

The outcome variables in this study encompass various aspects of PA, fitness self-assessment, school-related factors, BMI, and BMI categories according to the German cut-offs [20,21]. These included the school grade in physical education, which reflected the pupil’s performance in physical education. A key variable was positive self-assessment of fitness, where participants indicate whether the pupils considered themselves fit (yes or no). Additionally, swimming ability for 100 m was assessed, with categories including “Yes, but not so good”, “Yes, very good”, or “No”. Steps per day, an indicator considered relevant to measure daily PA in children [22], was used in our study to classify participants in categories such as “Do not track”, “<5000 steps”, “<10,000 steps”, and “>10,000 steps”. The study also evaluated the time spent on PA per day and included categories including <1 h, 1 h, 1–2 h, and >2 h. For high-intensity PA, time spent on high-pulse PA per week was categorized by frequency (<1 time, 1 time, 2–3 times, and >3 times), as was time spent on muscular PA per week (with similar frequency groupings). These outcome variables provide a comprehensive view of participants’ PA levels, fitness perceptions, and their involvement in physical education and sports.

### 2.5. Statistical Analysis

The characteristics of the study population such as age (in years), school level, and BMI was compared between sex categories. Key outcome variables, including sports grade, self-assessed fitness, swimming ability, and PA behaviors (steps per day, time spent on PA per day, and time spent on high-pulse and muscular PA per week) were summarized and compared between males versus females. To examine group differences for continuous variables Student’s *t* tests were performed and to evaluate differences between proportions of categorical variables chi-square tests were used. Univariate logistic regression models were employed to explore the relationship between sex and binary outcomes. Linear regression was used to quantify changes in mean BMI by sport club membership and type of sports club versus membership to no sports club. Multivariable logistic models were adjusted for age. Effect sizes were reported where relevant (odds ratios with 95% confidence intervals). Statistical significance was set at an alpha level of 0.05. All analyses were conducted using R version 4.4.2 (2024-10-31 ucrt) [23].

## 3. Results

### 3.1. Study Population

Of the total enrolled pupils as of 31 December 2024 (*n* = 922), 883 pupils aged 8 to 18 years were included. The characteristics of the included pupils is summarized in Table 1. The mean (SD) age of the included pupils was 13.1 ± 2.4 years. Around 46% (*n* = 406) were females and 54% (*n* = 477) were boys. Included pupils had a mean (SD) BMI of 19.4 ± 3.7 kg/m^2^. Weight status based on BMI categories did not differ by sex. The average school grade in physical education was 1.5 and 5.4% of the pupils did not consider themselves fit.

### 3.2. Sex Differences in Physical Activity

Regarding sex differences, no significant differences were found in age, BMI, sports grade, school level, or positive self-assessment of fitness. However, PA indicators differed by sex (Table 1). For example, a lower proportion of females (57.6%) could swim 100 m as compared to males (72.1%). Interestingly, nearly 6% of all pupils (females: 7.6%, males: 4.4%) could not swim. We found that one in two pupils tracked their daily steps and 17% males had more than 10,000 steps per day (females: 11.1%). With regard to PA per day, females were twice as likely to spend less than 1 h per day on PA than males (females: 21.7%, males: 11.9%). Females were half as likely to spent more than 2 h on PA than males (females: 9.4%, males: 18%). Time spent on high-pulse PA per week was also different between sex. While 27.9% males did high-pulse PA three times a week (females: 15.5%), 11.8% females spent less than 1 h per week on such activity (males: 7.5%). Although not statistically significant, a similar trend was observed in the time spent on muscular PA per week, with a lower proportion of females compared to males engaging in it.

**Figure 1:**
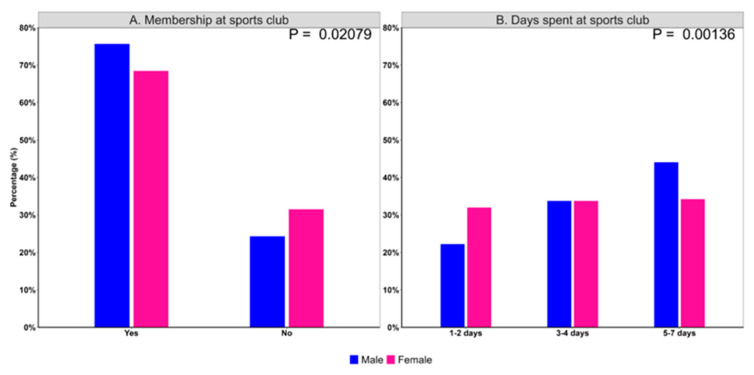
Sports club membership and attendance stratified by sex. Differences between sex groups were tested using χ2 test. (**A**) Proportion of sports club membership in males and females. (**B**) Proportion of days spent at sports club by males and females.

Next, it was assessed whether there were differences between sex groups and membership in sports clubs. The proportion of membership at sports club (*p* = 0.021) and more days spent at sports club (*p* = 0.0014) was higher in males than females (Figure 1).

In fact, as compared to females, males had a 43% higher chance of being members of a sport club (Table 2). Even after adjusting for age, this association remained significant (OR [95% CI]: 1.47 [1.09, 1.99]). Furthermore, males (compared to females) had a 44% higher chance of spending 3–4 days at the sports club and an 85% higher chance of spending 5–7 days there. After adjusting for age, these associations increased to 59% and doubled, respectively.

### 3.3. Differences in Sports Types Participated in by Sex

**Figure 2:**
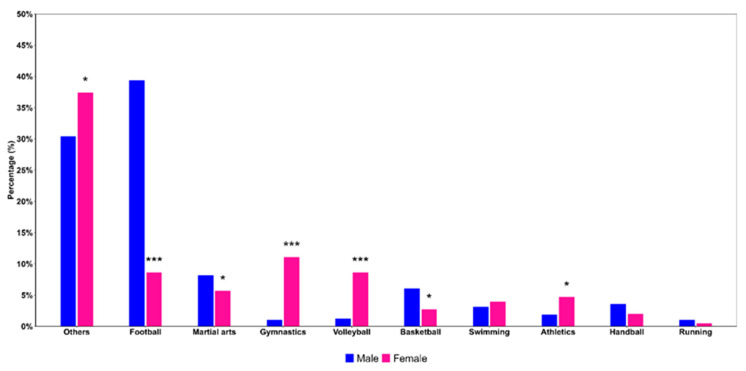
Sport activities stratified by type and sex. Differences between sex groups within each sport type were tested using the chi-square test. Statistically significant results are shown as *** (*p* < 0.001) and * (0.01 ≤ *p* < 0.05).

We further assessed proportion of males and females participating in different sports. As shown in Figure 2, a significantly higher proportion of males were involved in football (males versus females: 39.4% versus 8.6%, *p* < 0.00001), martial arts (males versus females: 8.2% versus 5.7%, *p* = 0.0265), and basketball (males versus females: 6.1% versus 2.7%, *p* = 0.02524). On the other hand, females were more involved in other sports, which included tennis and horse riding (males versus females: 37.4% versus 30.4%, *p* = 0.0327) than males. A higher proportion of females than males were also involved in gymnastics (males versus females: 1.0% versus 11.1%, *p* < 0.00001), volleyball (males versus females: 1.3% versus 8.6%, *p* < 0.00001), and athletics (males versus females: 1.9% versus 4.7%, *p* = 0.0302). No significant differences by sex were found in sports such as swimming, handball, or running.

### 3.4. Differences in Sports Types Participated in by Age

**Figure 3:**
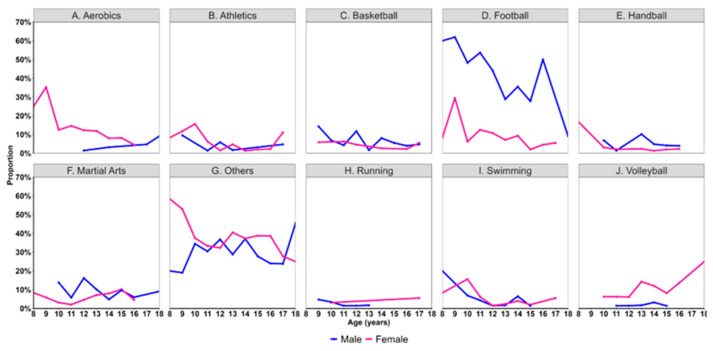
Sport activity participation by age and sex. (**A**) Participation in aerobics. (**B**) Participation in athletics. (**C**) Participation in basketball. (**D**) Participation in football. (**E**) Participation in handball. (**F**) Participation in martial arts. (**G**) Participation in other sport activities. (**H**) Participation in running. (**I**) Participation in swimming. (**J**) Participation in volleyball.

In the next step, the proportion of males and females participating in different sports by age was assessed (Figure 3). Differences in sport types participated in by age were not apparent for most of the sports. However, a drastic difference over age was found in football as the participation was always higher in males than females. While at 8 years of age, 60% males versus 8.3% females played football, the proportion reduced to 9.1% in males versus 5.6% in females by 18 years of age. Among males, a steep decline in football was noticed after age 16 years. An increasing age was associated with a 30% increased chance of absence of sports club membership in males (OR 1.30, 95% CI [1.18, 1.43]).

Among females, the uptake of aerobics already started before or at 8 years of age (25%) and slowly decreased to 4.5% by age 16 years. In contrast, a very low proportion of males were involved in aerobics after age 12 years. Starting at 10 years of age, a few females also played volleyball (6.3%). The proportion of males playing volleyball was ranged from 1 to 3.2% between ages 11 and 15. Already, at 8 years of age, nearly 58.3% females were involved in other sport activities (males: 20%). At 10 years of age, the proportion of uptake of other sport activities became similar in males (34.4%) and females (37.5%). However, at 18 years of age, more males (45.5%) were involved in other sport activities than females (25%). We observed that with age, females had a 13% higher likelihood of not being members of a sports club (OR 1.13, 95% CI [1.03, 1.24]).

### 3.5. Influence of Weight Status on Sports Club Membership

**Figure 4:**
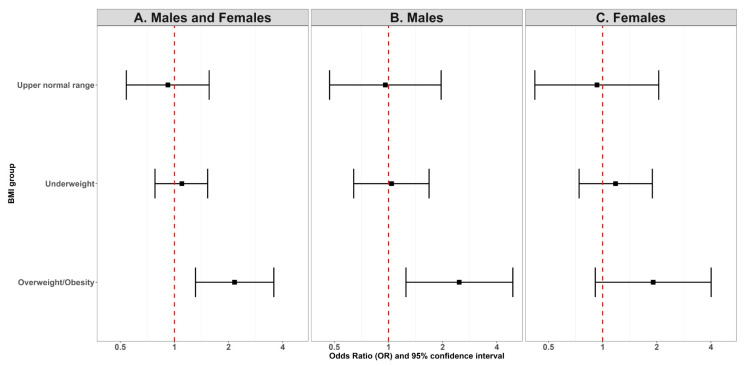
Membership to no sports club and weight status in pupils. Values are odds ratios (OR) and 95% confidence intervals based on univarite logistic regression, where normal weight pupils were considered as control group. The dashed line on the forest plots represents the point of no effect (OR = 1). (**A**) Odds ratios in males and females. (**B**) Odds ratios in males. (**C**) Odds ratios in females.

To evaluate the influence of weight status on sports club membership (yes versus no), we calculated the odds ratio of the club membership according to the different BMI groups and considered normal weight pupils as control (Figure 4). The likelihood of sport club membership did not differ between pupils who were a normal weight or underweight or had a BMI in the upper normal range, regardless of sex. However, pupils who were overweight or obese had a 2-fold increased risk of absence of sports membership as compared to normal weight pupils (OR [95% CI]: 2.16 [1.31, 3.57]). Even within the subgroup of males, pupils who were overweight or obese were at a 2.5-fold chances of having no sports club membership as compared to their counterparts who were normal weight. Such association was not found in the subgroup of females (OR [95% CI]: 1.91 [0.91, 4.01], *p* = 0.0889).

### 3.6. Type of Sports Club Membership and Weight Status

**Figure 5:**
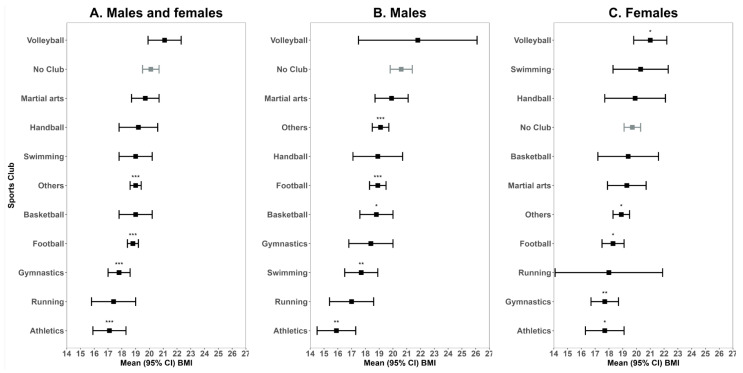
Mean (95%) BMI in pupils by type of sport club membership. Mean values are compared against the control group “No Club” (gray) indicating no membership to any sports club. Significant differences are shown as *p* < 0.0001 (***), *p* < 0.001 (**), or *p* < 0.05 (*) and are based on univariate linear regression models. (**A**) Odds ratios in males and females. (**B**) Odds ratios in males. (**C**) Odds ratios in females.

As the final step, we assessed the change in BMI based on type of sport club membership compared to absence of sport club membership (Figure 5). Among all pupils who did not have a membership to sports club, the mean BMI was 19.1 kg/m^2^ (95% CI 18.9, 19.3 kg/m^2^) which was not statistically different from pupils who were members of volleyball, martial arts, handball, swimming, basketball, or running sports clubs. However, compared to non-members, a significant lower mean BMI was observed in pupils who were members of athletics (mean 17.1 kg/m^2^, 95% CI [15.9, 18.3 kg/m^2^]), gymnastics (mean 17.8 kg/m^2^, 95% CI [17.0, 18.6 kg/m^2^]), football (mean 18.8 kg/m^2^, 95% CI [18.4, 19.2 kg/m^2^]), and other (mean 19.0 kg/m^2^, 95% CI [18.6, 19.4 kg/m^2^]) sport clubs. The highest difference in mean BMI was observed in all pupils who engaged in athletics versus pupils who did not perform any sports (mean difference in BMI in kg/m^2^: −2.98 [95% CI: −4.43, −1.53]).

We repeated the analysis in subgroups of males and females. Among males, we found that the mean BMI for pupils who did not have any sport club membership was 20.6 kg/m^2^. Compared to male non-members, lower mean BMI levels were found in counterpart males who played football (mean 18.9 kg/m^2^, 95% CI [18.3, 19.5 kg/m^2^]), basketball (mean 18.8 kg/m^2^, 95% CI [17.6, 20.0 kg/m^2^]), swimming 17.7 kg/m^2^, 95% CI [16.5, 18.9 kg/m^2^]), or athletics (mean 15.9 kg/m^2^, 95% CI [14.5, 17.3 kg/m^2^]). The largest mean BMI difference was observed between males who participated in athletics and those who did not engage in any sports, with a mean difference of −4.64 kg/m^2^ (95% CI: 7.31, −1.98).

In the subgroup of females, those who did not participate in any sports had a mean BMI of 19.7 kg/m^2^, 95% CI [19.1, 20.3 kg/m^2^], which was even lower than females who played volleyball (mean 21.0 kg/m^2^, 95% CI [19.8, 22.2 kg/m^2^]). Additionally, in contrast to females who were not members of sport club, a higher mean BMI was found in females who engaged in football (mean 18.3 kg/m^2^, 95% CI [17.5, 19.1 kg/m^2^]), gymnastics (mean 17.7 kg/m^2^, 95% CI [14.5, 17.3 kg/m^2^]), athletics (mean 17.7 kg/m^2^, 95% CI [16.3, 19.1 kg/m^2^]), or other sport (mean 18.9 kg/m^2^, 95% CI [18.3, 19.5 kg/m^2^]) activities. The most significant mean BMI difference was also found between females who took part in athletics and those who did not participate in any sports, with a mean difference of −1.99 kg/m^2^ (95% CI: −3.65, −0.36).

## 4. Discussion

In this large study of more than 800 school-aged children and adolescents, we examined the participation rates of males and females in various sports. Our study reveals notable sex-based differences in sports involvement. Male pupils were 50% more likely to be members of sports club and spend more time on sport activities than their female counterparts. Furthermore, males were largely involved in contact sports such as football, martial arts, and basketball while females engaged more often in gymnastics, athletics, volleyball, and other sports activities including horse riding. Although the uptake of sports generally varied with age, involvement in sports was related to better weight status in pupils as opposed to pupils who did not perform any sports. The highest impact on weight status was observed when pupils were engaged in athletics, regardless of sex.

Engagement in sport activities is a crucial factor of physical health and overall well-being. Several previous studies have shown that early participation in sports has a lasting impact on PA levels, mental health, and social development [24,25]. However, sport participation can vary between sex and across age. A recent study of 123,809 adults from 28 European countries showed that a number of persons not fulfilling PA guide-lines increased over the past 17 years and there were no major differences in males and females [26]. Another study comparing the distribution of PA levels between 15,461 boys and girls aged 5–18 years in nine different countries found that boys were engaged in greater activity levels than girls, which aligns with the findings of our study [16]. Nonetheless, the type of sport activity performed was not evaluated in this study. A comparatively smaller study including 364 Spanish children aged 7–11 years evaluated the sex-specific engagement in different sports [27]. This study aligns with our findings, showing that males are consistently more likely to engage in organized sports, especially contact sports like football and basketball. One possible reason is that contact sports are often more popular and accessible for boys, due to societal norms that link masculinity with physicality and competition [28], which we hypothesize could influence behaviors from childhood onwards. On the other hand, females tend to prefer non-contact sports or those seen as more “feminine”, such as gymnastics, volleyball, and dance, as shown in a recent study [29]. We assume that from a young age, girls are taught to value qualities like grace, flexibility, and coordination, which are often associated with sports such as gymnastics, volleyball, and dance.

The gender gap in sports participation becomes especially evident during adolescence, as social pressures, changing interests, and developmental shifts impact children’s involvement in physical activities [30]. In our study, apart from engagement in volleyball in females and other sports in males, we observed a reduction in sport activities over age, regardless of gender. As also noted previously, adolescents may reduce sports participation due to physical changes during puberty [31], which can affect self-esteem and coordination [32]. Social pressures, particularly around body image, discourage girls from engaging in physical activities [33]. It may also be possible that academic demands and extracurricular activities can often take precedence over sports during adolescence.

Recent studies have examined the relationship between weight status and sports club membership among children, highlighting significant associations. A recent study on more than 9000 adolescents examined the impact of health club membership on cardiovascular health and sedentary behavior. The study found that participants with health club memberships had lower resting heart rates, improved cardiorespiratory fitness, and reduced sedentary time compared to non-members [15]. While engaging in sports can influence better weight status in children and adolescents [34,35], present weight status can also influence participation in sports. In our study, we found that pupils with overweight/obesity were twice as likely to not be members of sport club as opposed to normal-weight counterparts. Another study found that there was a 16–19% increased chance of non-membership to sports club among overweight/obese subjects aged 6 to 15 years [36]. Children with overweight and obese children face several barriers to sports participation, including physical, psychological, and social factors [37,38]. Physically, overweight/obese children may have lower endurance and fear of injury, reducing their confidence in sports. Psychologically, body image issues and fear of stigma discourage participation. Socially, a lack of support from peers or family can lead to feelings of exclusion, further lowering participation and worsening obesity-related health risks [39]. In order to encourage sports participation in overweight and obese children, public health programs should prioritize fun, body positivity, and inclusivity. Activities should cater to various fitness levels, with family and peer involvement offering support [40,41].

The analysis of weight (BMI) changes based on different sports club membership reveals important insights into the relationship between PA and weight status in children. While no significant differences were found in BMI between pupils who were members of volleyball, martial arts, handball, swimming, basketball, or running sports clubs and those who did not participate in any sports, notable reductions in BMI were observed among pupils involved in athletics, gymnastics, football, and other sport clubs. Specifically, an athletics membership was associated with the most significant reduction in BMI, with a mean difference of 2.98 kg/m^2^, suggesting that high-intensity, endurance-based sports might have a more pronounced impact on weight status compared to other activities. These findings are consistent with previous research indicating that aerobic exercises, such as those performed in athletics, contribute to greater calorie expenditure and fat reduction, leading to lower BMI [41]. Gymnastics, football, and other team-based sports also showed reduced BMI, though the differences were smaller compared to athletics, which could reflect the varying levels of intensity and energy expenditure inherent in different sports. While sports participation generally has a positive effect on BMI, it is clear that the type of sport matters. This underscores the importance of encouraging children to engage in a variety of physical activities, particularly those with a higher intensity, to achieve the best outcomes in terms of weight management and overall health.

This large German study, which includes boys and girls aged 8–18 years in a school setting, provides valuable insights into the relationship between BMI and sports participation. Its strengths lie in the large sample size, which enhances the reliability and generalizability of the findings, as well as the inclusion of both genders, allowing for gender-based comparison. The study’s focus on different sports and BMI measurements in both males and females offers a comprehensive view of how PA influences weight status across various groups. Understanding these differences helps in promoting the most effective sports for each gender, ensuring better health outcomes for both. However, there are limitations, including the lack of consideration for factors such as socioeconomic status, family support, or psychological barriers that could also influence sports participation. Additionally, the study does not differentiate between the types of sports in terms of intensity or frequency, missing the opportunity to explore how various sports might affect BMI differently. Furthermore, the absence of longitudinal data limits the ability to assess the long-term effects of sports participation on BMI changes over time. Lastly, children below the age of 8 years were excluded, as fluent reading was required to complete the questionnaire. Future studies should focus on exploring the impact of different types of sports, accounting for socioeconomic, psychological, and environmental factors, and include longitudinal data to assess long-term effects on BMI and overall health, while also examining strategies to increase participation among underrepresented groups.

## 5. Conclusions

In conclusion, addressing gender gaps in sports participation is crucial, with a particular focus on encouraging females and overweight/obese males to engage more in physical activities. Both traditionally male and female sports, in general, should be made accessible to all children and adolescents, promoting inclusivity. Offering a diverse range of activities can lead to increased sports participation, resulting in long-term health benefits.

## Figures and Tables

**Table 1:** Characteristics and sport activities of the Hand-on-Heart-study population.

Characteristics and Sport Activities	All Pupils	Female	Male	*p*-Value
	*N* (%)	*n* (%)	*n* (%)	
Number of pupils	883	406	477	
Age, years	13.1 ± 2.4	13.0 ± 2.3	13.1 ± 2.4	0.704
Age categories, years				0.323
8	27 (3.1)	15 (3.1)	12 (3.0)	
9	38 (4.3)	21 (4.4)	17 (4.2)	
10	61 (6.9)	29 (6.1)	32 (7.9)	
11	117 (13.3)	69 (14.5)	48 (11.8)	
12	133 (15.1)	68 (14.3)	65 (16.0)	
13	101 (11.4)	59 (12.4)	42 (10.3)	
14	137 (15.5)	62 (13.0)	75 (18.5)	
15	121 (13.7)	72 (15.1)	49 (12.1)	
16	94 (10.6)	50 (10.5)	44 (10.8)	
17	39 (4.4)	21 (4.4)	18 (4.4)	
18	15 (1.7)	11 (2.3)	4 (1.0)	
Body mass index (BMI), kg/m^2^	19.4 ± 3.7	19.4 ± 3.5	19.4 ± 3.8	0.901
BMI categories				0.487
Underweight	301 (34.2)	137 (33.8)	164 (34.5)	
Normal weight	411 (46.7)	198 (48.9)	213 (44.8)	
Weight in the upper normal range	91 (10.3)	36 (8.9)	55 (11.6)	
Overweight/Obesity	77 (8.8)	34 (8.4)	43 (9.1)	
School grade in physical education	1.51 ± 0.70	1.53 ± 0.70	1.49 ± 0.70	0.442
School level				0.258
Primary School	100 (11.3)	43 (10.6)	57 (11.9)	
Middle School	58 (6.6)	21 (5.2)	37 (7.8)	
Secondary School	604 (68.4)	290 (71.4)	314 (65.8)	
High School	121 (13.7)	52 (12.8)	69 (14.5)	
Positive self-assessment of fitness				0.670
Yes	835 (94.6)	382 (94.1)	453 (95.0)	
No	48 (5.4)	24 (5.9)	24 (5.0)	
Swim for 100 m				<0.001
Yes, but not so good	253 (28.7)	141 (34.7)	112 (23.5)	
Yes, very good	578 (65.5)	234 (57.6)	344 (72.1)	
No	52 (5.9)	31 (7.6)	21 (4.4)	
Steps per day				<0.001
Do not track	477 (54.0)	211 (52.0)	266 (55.8)	
<5000	40 (4.5)	29 (7.1)	11 (2.3)	
<10,000	240 (27.2)	121 (29.8)	119 (24.9)	
>10,000	126 (14.3)	45 (11.1)	81 (17.0)	
Time spent on PA/day				<0.001
<1 h	145 (16.4)	88 (21.7)	57 (11.9)	
1 h	246 (27.9)	126 (31.0)	120 (25.2)	
1–2 h	368 (41.7)	154 (37.9)	214 (44.9)	
>2 h	124 (14.0)	38 (9.4)	86 (18.0)	
Time spent on high-pulse PA/week				<0.001
<1 time	84 (9.5)	48 (11.8)	36 (7.5)	
1 time	178 (20.2)	107 (26.4)	71 (14.9)	
2–3 times	425 (48.1)	188 (46.3)	237 (49.7)	
>3 times	196 (22.2)	63 (15.5)	133 (27.9)	
Time spent on muscular PA/week				0.085
<1 time	226 (25.6)	100 (24.6)	126 (26.4)	
1 time	274 (31.0)	141 (34.7)	133 (27.9)	
2–3 times	284 (32.2)	128 (31.5)	156 (32.7)	
>3 times	99 (11.2)	37 (9.1)	62 (13.0)	

Data are mean ± SD or *n* (%) and tested with regard to the sex using Student’s *t* test for continuous and χ2 test for categorical variables.

**Table 2:** Club membership and activity by sex.

Outcome	*N*	Unadjusted	Age-Adjusted
Sports club membership			
No	244	Reference	Reference
Yes	639	**1.43 (1.07, 1.93)**	**1.47 (1.09, 1.99)**
Days spent at sports club			
1–2 days	236	Reference	Reference
3–4 days	298	**1.44 (1.02, 2.03)**	**1.59 (1.11, 2.26)**
5–7 days	349	**1.85 (1.33, 2.59)**	**2.00 (1.37, 2.91)**

Values are odds ratios and 95% confidence intervals based on logistic regression. The influence of sex (Control versus Test: females versus males) was assessed for each outcome. Bold text indicates *p* < 0.05.

## Data Availability

Data cannot be shared publicly because participants did not provide explicit consent for data sharing in accordance with the European Union’s General Data Protection Regulation and relevant German privacy laws. However, data can be made available to researchers who meet the criteria for access to confidential information through the Research Ethics Board of Ludwig-Maximilians-Universität Munich, Germany. Requests should be directed to: ethikkommission@med.uni-muenchen.de.

## Abbreviations

The following abbreviations are used in this manuscript:

BMI	Body mass index
OR	Odds ratio
PA	Physical Activity
SD	Standard deviation
WHO	World health organization
